# Modelling Runoff and Sediment Loads in a Developing Coastal Watershed of the US-Mexico Border

**DOI:** 10.3390/w11051024

**Published:** 2019

**Authors:** Napoleon Gudino-Elizondo, Trent W. Biggs, Ronald L. Bingner, Eddy J. Langendoen, Thomas Kretzschmar, Encarnación V. Taguas, Kristine T. Taniguchi-Quan, Douglas Liden, Yongping Yuan

**Affiliations:** 1 Department of Civil and Environmental Engineering, University of California, Irvine, CA 92697, USA; 2 Department of Geography, San Diego State University, 5500 Campanile Dr., San Diego, CA 92182-4493, USA;; 3 Departamento de Geología, Centro de Investigación Científica y de Educación Superior de Ensenada (CICESE), Carretera Ensenada-Tijuana 3918, Zona Playitas, 22860 Ensenada, B.C., Mexico;; 4 National Sedimentation Laboratory, Agricultural Research Service, USD A, Oxford, MS 38655, USA;; 5 Department of Rural Engineering, University of Córdoba, Córdoba, 14071, Spain;; 6 Southern California Coastal Water Research Project, 3535 Harbor Boulevard, Suite 110, Costa Mesa, CA 92626, USA;; 7 USEPA San Diego Border Liaison Office, 610 West Ash St., Suite 905, San Diego, CA 92101, USA;; 8 USEPA Office of Research and Development, Research Triangle Park, NC 27711, USA;

**Keywords:** soil erosion, rainfall-runoff, sediment yield, AnnAGNPS model, urbanization, scenario analysis

## Abstract

Urbanization can increase sheet, rill, gully, and channel erosion. We quantified the sediment budget of the Los Laureles Canyon watershed (LLCW), which is a mixed rural-urbanizing catchment in Northwestern Mexico, using the AnnAGNPS model and field measurements of channel geometry. The model was calibrated with five years of observed runoff and sediment loads and used to evaluate sediment reduction under a mitigation scenario involving paving roads in hotspots of erosion. Calibrated runoff and sediment load had a mean-percent-bias of 28.4 and − 8.1, and root-mean-square errors of 85% and 41% of the mean, respectively. Suspended sediment concentration (SSC) collected at different locations during one storm-event correlated with modeled SSC at those locations, which suggests that the model represented spatial variation in sediment production. Simulated gully erosion represents 16%–37% of hillslope sediment production, and 50% of the hillslope sediment load is produced by only 23% of the watershed area. The model identifies priority locations for sediment control measures, and can be used to identify tradeoffs between sediment control and runoff production. Paving roads in priority areas would reduce total sediment yield by 30%, but may increase peak discharge moderately (1.6%–21%) at the outlet.

## 1 Introduction

Erosion, defined as the detachment, transport, and spatial redistribution of soil particles [1,2], contributes to environmental degradation around the globe [3]. Urbanization can lead to an increase scenario analysis in erosion and the discharge of terrigenous materials into downstream ecosystems, including inland lakes, reservoirs, estuaries, and oceans.

Sheetwash, rill, and gully erosion, hereafter referred to as hillslope erosion, are frequently associated with anthropogenic soil disturbance and are often related to land use change, such as deforestation and urban development [4,5]. Hillslope erosion processes have been well characterized in agricultural settings, but not in urbanized areas where high erosion rates have also been reported [6]. Hillslope erosion rates typically decrease as bare soil in construction sites is replaced by impervious surface and vegetation [7]. Conversely, in developing countries, soil exposure such as vacant lots and unpaved roads can persist for longer periods [8], which increases hillslope erosion rates compared to other urban watersheds with high impervious cover fractions [9] and storm-water management practices [10].

Hillslope erosion and sediment production can be simulated using numerical models that consider the relationship between terrain attributes and climate regimes [11,12]. These models vary in structure, assumptions, and data requirements [13,14]. Erosion modeling is often used to simulate various erosional processes, such as sheet, rill, gully, and channel erosion, to develop sediment budgets and to assess the effect of Best Management Practices (BMPs) on total sediment reduction. The sediment budget is the quantitative tracking of contributing sources, sinks, and spatial redistribution of sediments over a given time scale [15].

Several soil erosion studies have focused on sheet and rill processes [16–19], but ephemeral gullies can also contribute a significant source of sediment at the catchment scale [20–22], especially in arid and semi-arid areas [23]. Such gullies are caused by concentrated overland flow [24] and are commonly cleared by tillage operations [25] or, in urban environments, filled with unconsolidated sediment during grading [9]. These erosional features form due to a complex relationship between terrain and management characteristics such as slope, land cover, soil properties, climate regime, and management activities [26].

The Annualized Agricultural Non-Point Source (AnnAGNPS) model is a simulation tool developed by the USD A-Agricultural Research Service and the Natural Resources Conservation Service (NRCS) to evaluate the effect of land use and management activities on watershed hydrology and sediment transport [12]. AnnAGNPS simulates runoff and sediment generation by tracking their transport through the channel network (AnnAGNPS reaches) at the watershed scale on a daily time step. AnnAGNPS simulates different erosional processes (i.e., sheet, rill, and gullies) as well as channel sources. Sheet and rill erosion are simulated using the Revised Universal Soil Loss Equation (RUSLE). Ephemeral gully erosion is simulated using EGEM (Ephemeral Gully Erosion Model) whose hydrologic routines to calculate peak and total discharge are estimated following the SCS curve number (CN) methodology [27], and gully width and soil erosion calculations are based on the Chemical, Runoff, and Erosion from the Agricultural Management Systems (CREAMS) model [28]. The model simulates colluvial storage of sediment using the Hydro-geomorphic Universal Soil Loss Equation (HUSLE), which calculates a delivery ratio based on particle size distribution and flow transport capacity [29].

The AnnAGNPS model has been tested in small Mediterranean watersheds (< 1.3 km^2^) [14,23,30]. Licciardello et al. [30] evaluated AnnAGNPS in a steep catchment under pasture in Eastern Italy. Taguas et al. [23] evaluated the effect of different management activities on total sediment reduction in an agricultural environment in Spain, where ephemeral gullies are a significant contributor to the total sediment production. Gudino-Elizondo et al. [14] reported good performance of AnnAGNPS in simulating ephemeral gullies at the neighborhood scale in an urban watershed. However, AnnAGNPS has not been tested to model hillslope erosion rates in an urbanizing catchment under different soil types and land uses.

This study aims to (1) test the capabilities of AnnAGNPS to simulate runoff and sediment production in an urban watershed in a developing country context and (2) use the model to constrain the sediment budget in order to inform management and policy designed to mitigate sediment loads downstream. This paper addresses the following research questions: (a) How accurately does AnnAGNPS simulate water and sediment loads in an urban watershed in a developing-country context where ephemeral gullies are likely to be a significant source of sediment? (b) What processes generate sediment in the watershed, and what is the role of soil properties and land use? (c) How does storm size affect the sediment load from different hillslope processes (sheet and rill, and gully erosion)? (d) Where are hot spots of sediment production, and what watershed characteristics control sediment production? and (e) What are the implications of the sediment budget and distribution of hotspots for management designed to mitigate sediment loads?

## 2. Materials and Methods

### 2.1. Study Area

**Figure 1.**
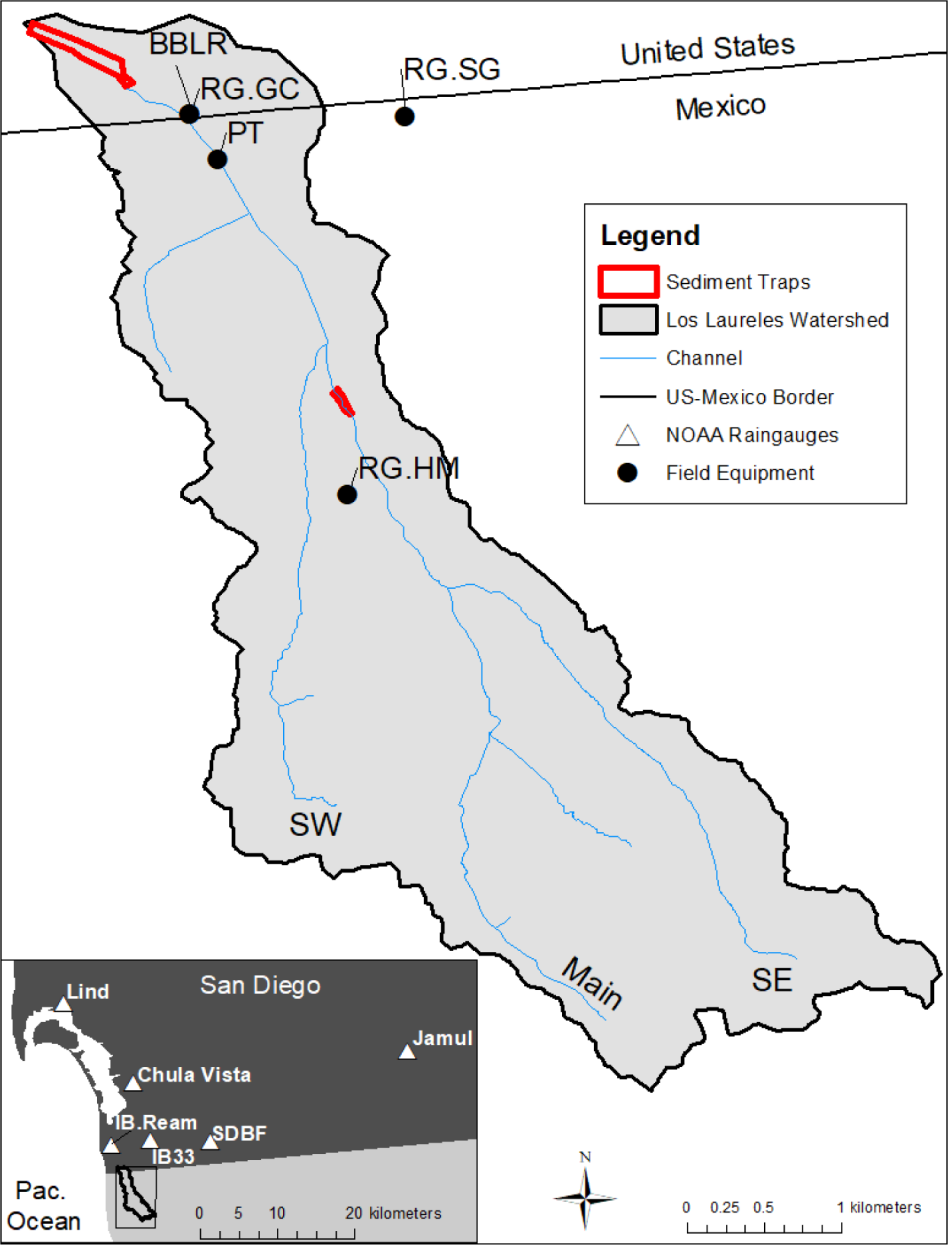
Location of the Los Laureles Canyon Watershed, main channels (SW, Main, and SE), and monitoring stations including sediment traps and rain gauges (RG). Inset shows the geographic locations of nearby rain gauges used in this analysis to span the rainfall time series.

The Los Laureles Canyon Watershed (LLCW) is a transboundary urbanizing catchment located in the northwestern part of Tijuana, Mexico, which flows into the Tijuana River Estuarine Reserve, USA (Figure 1). The total catchment area is 11.6 km^2^, with 93% in Mexico and 7% in the USA. The climate in LLCW is Mediterranean, with a rainy season during the winter and annual mean rainfall of approximately 240 mm. Most of the erosional storm events occur during the winter. The regional geology (San Diego formation) includes marine and fluvial sediment deposits of conglomerate, sandy conglomerate, and siltstone. Soils are sandy with a wide range of cobble fraction, and are dominated by abrupt slopes (15°, average), which encourages gully formation and results in high erosion rates.

The LLCW is an uplifted and incised marine terrace, where the soil types are controlled in part by the underlying geology. The conglomerate geology in the northern part of the watershed has steep, competent valley walls with relatively flat buttes and mesas. In the central part of the watershed, the sandy conglomerate geology with low cobble fraction has lower slopes and rounded hilltops. The southern part of the watershed is a relatively flat, non-incised conglomerate. A narrow valley floor has Quaternary alluvium, but most of this has been paved or channelized.

Land use in the LLCW is predominantly mixed urban and rural, and was urbanized starting in 2002 with many illegal housing developments (“invasions”). Gullies form on unpaved roads, which affect civil infrastructure in the upper watershed [31] and downstream ecosystems [32]. Such gullies are filled in with sediment following storms, and this management practice should be taken into account in developing the sediment budget and in soil erosion modeling for the watershed.

Sediment from the LLCW has buried native vegetation in the Tijuana River Estuary, which is located downstream of LLCW in the United States. In response, sedimentation basins were constructed at the outlet in the US in 2004, which costs $3 million USD to clean annually [32]. Stormflow and erosion also threaten human life, which causes damage to roads and houses in Tijuana [31]. The primary sources of sediment from LLCW are gully formation on unpaved roads, channel erosion, and sheet and rill erosion from unoccupied lots in Tijuana [8].

### 2.2. Field Data Collection and Model Setup

A summary of the data collection activities is reported in Reference [33]. Briefly, a tipping-bucket rain gauge station (RG.HM in Figure 1) was installed in February 2013. A pressure transducer (PT) (Solinst, water level logger) was installed in a concrete channel at the watershed outlet in December 2013 to record the water stage at 5-minute intervals (Figure 1). The stage-discharge relationship was determined using Manning’s equation and flow velocity measurements. Manning’s roughness coefficient (n) was based on field measurements of discharge in 2016 and 2017, which was used to back-calculate a Manning’s *n*. The discharge measurements were also used to create a stage-discharge relationship for a stream gauge in the US (RG.GC) to complete our observations when the pressure transducer malfunctioned. The PT data were also validated and supplemented using time-lapse photographs of the water stage at the PT station. Suspended Sediment Concentration (SSC) measurements were taken at 10 different locations during a storm on 27 February, 2017 to explore spatial patterns of sediment production within the watershed. Annual sediment load data was collected in two large sediment traps at the watershed outlet. Data on the quantity of sediment removed annually (2006–2012) from the traps were available from the Tijuana River National Estuarine Research Reserve (TRNERR), corrected for trap efficiency, and used for model calibration (Figure 1).

A map of soils and associated parameters needed for the model were not available for the watershed, so soil type and parameters were mapped by modifying an existing geology map [34] and a correlation between geology and soil type that was established using the Soil Survey Geographic Database (SSURGO) from the United States Department of Agriculture. The geology map was modified based on field data collected during September 2015 and visual interpretation of high resolution imagery on Google Earth. First, a seamless cross-border geological map was created using the Instituto Metropolitano de Planeación de Tijuana, Baja California Mexico (IMPLAN) [34] geology map for Mexico and a geology map from the US [35]. The US soils that occurred on geologic types found in LLCW were identified as candidate soils for the study watershed. See Biggs et al. [33] for a full description of field and laboratory data collection.

**Figure 2.**
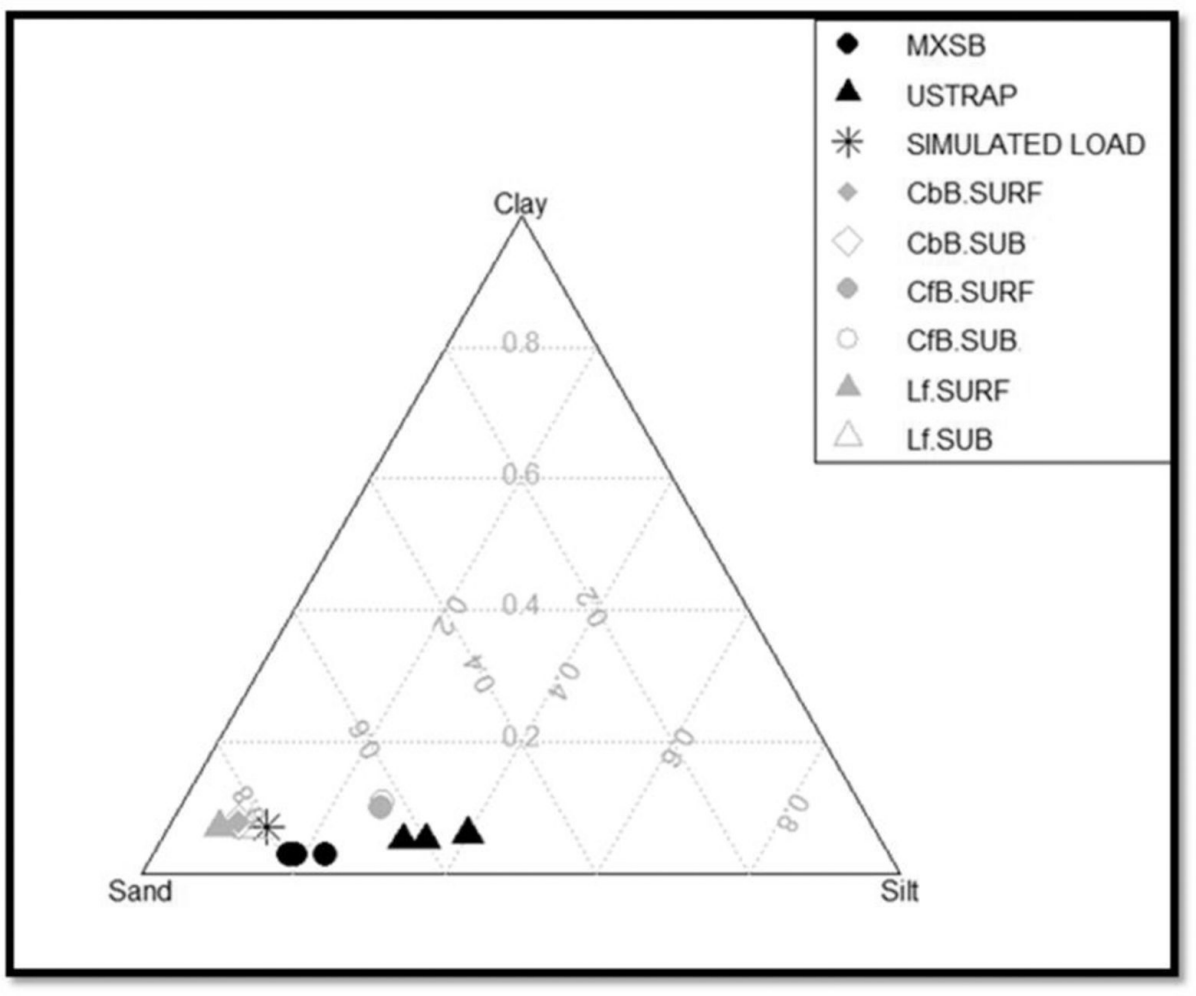
Ternary diagram of the mean grain sizes observed in the surface (SURF) and subsurface (SUB > 50 cm depth) soil layers for each soil type in the watershed, and in the sediment traps in Mexico (MXSB) and at the outlet in USA (USTRAP).

**Figure 3.**
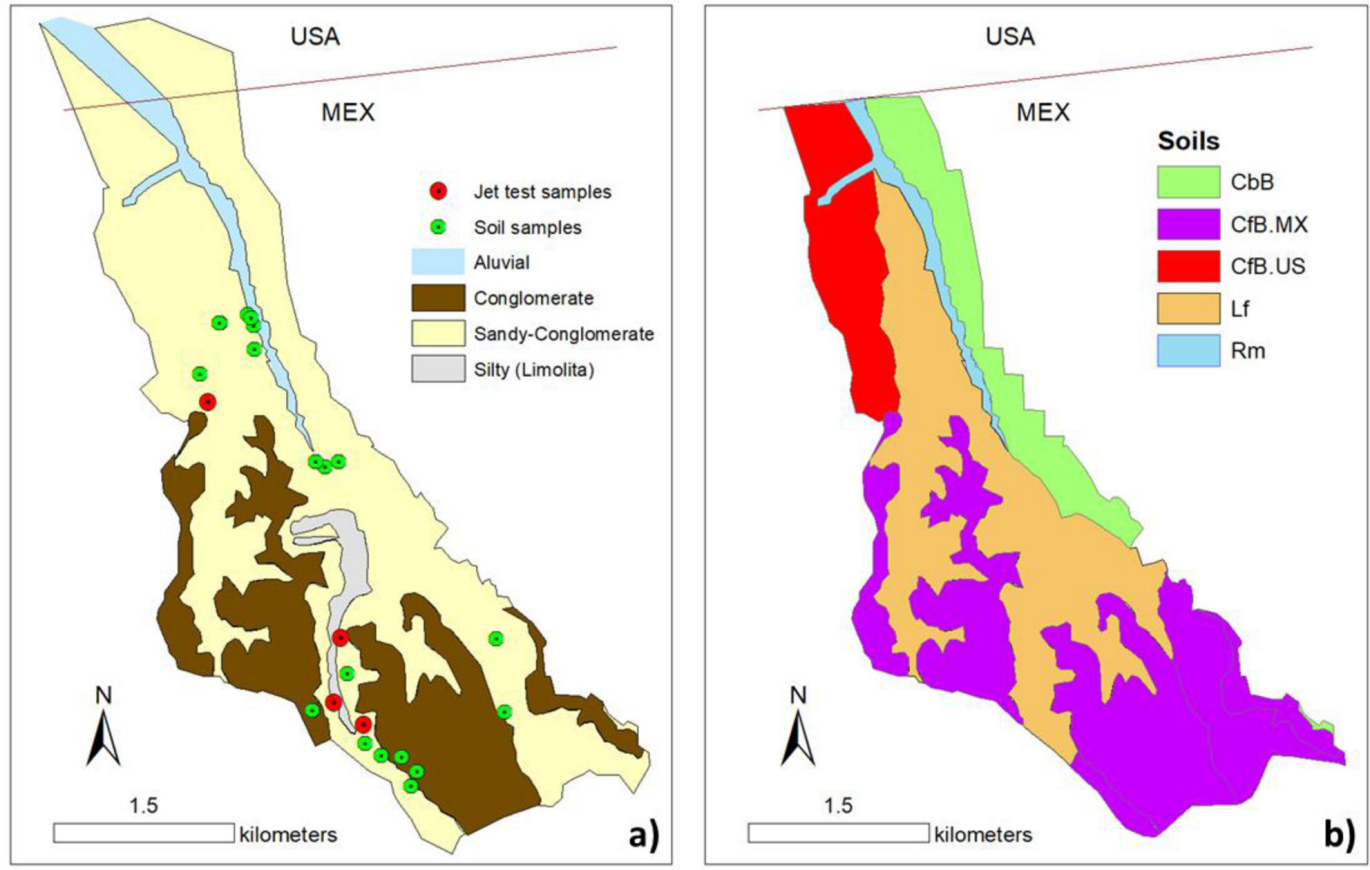
Geology map **(a)** and updated soils map for LLCW **(b)**. Las Flores (Lf), fine sandy loam, dominates the central portion of the watershed (orange). CfB.MX represents the Chesterton sandy loam (CfB), but with a cobbly surface horizon. Carlsbad (CbB) and “CfB.US” soils extend south from the US/Mexico border.

Three main soil types were identified: (1) Los Flores formation (Lf) is a loamy fine sand, (2) the Chesterton formation (CfB) is conglomerate dominant with a fine sandy loam matrix, and (3) the Carlsbad formation (CbB) is a gravelly loamy sand. Once candidate soils were determined from the US geology and soils maps, the SSURGO soil characteristics were extracted for all horizons for comparison with data collected in the field. Soil samples were then collected from different geology types and analyzed for texture to compare with the SSURGO database and with the observed soil texture in the sediment traps at the watershed outlet (Figure 2). Samples *(N* = 25) were collected from road cuts and other exposed profiles from the near-surface (10–50 cm) and from the subsurface (>50–100 cm). The cobble percentage was determined through point counts along aim transect through each distinct horizon. A bulk sample of sediment smaller than coarse gravel (<32 mm) was collected for texture analysis, analyzed in the laboratory using dry sieving to separate a 2-mm fraction and the pipette method for fines (<2 mm). Soil texture for all soil samples collected in LLCW and near the US-Mexico border was plotted in ternary diagrams and compared to SSURGO surface and subsurface soil texture (Figure 2). For each soil group, the SSURGO soil types that most closely matched the mean texture from the soil samples, were selected and used to update the soils map for LLCW. For some areas, the texture from the samples was similar to SSURGO data (northern part of the watershed). For the CfB soils, the texture from the samples did not match the SSURGO texture (southern part of the watershed), so a new geologic type (CfB.MX) was created, supported by field and laboratory data, to include in the AnnAGNPS model. Lastly, polygons delineating soil types were created by first determining the relationship between soil color, landform, and soil type for soils in the US, and then extrapolating those relationships to map similar soils in the LLCW (Figure 3).

The critical shear stress (*τ*_*c*_) and soil erodibility in the non-cobbly sandy conglomerate (Lf) were taken from Gudino-Elizondo et al. [14], who used a mini-jet erosion test device following the methodology described by Hanson [36]. Values of *τ*_*c*_ for conglomerate soil were initially taken from USGS [37] and were modified during calibration.

The AnnAGNPS model requires daily precipitation data. To extend the simulation period to include the period after the installation of the sediment trap (2004–2017), the precipitation data collected from February 2013 to 2017 at RG.HM were compared with rainfall data from nearby stations in the United States (Figure 1) to select the best rain gauge to use for rainfall data from 2004–2013.

**Figure 4.**
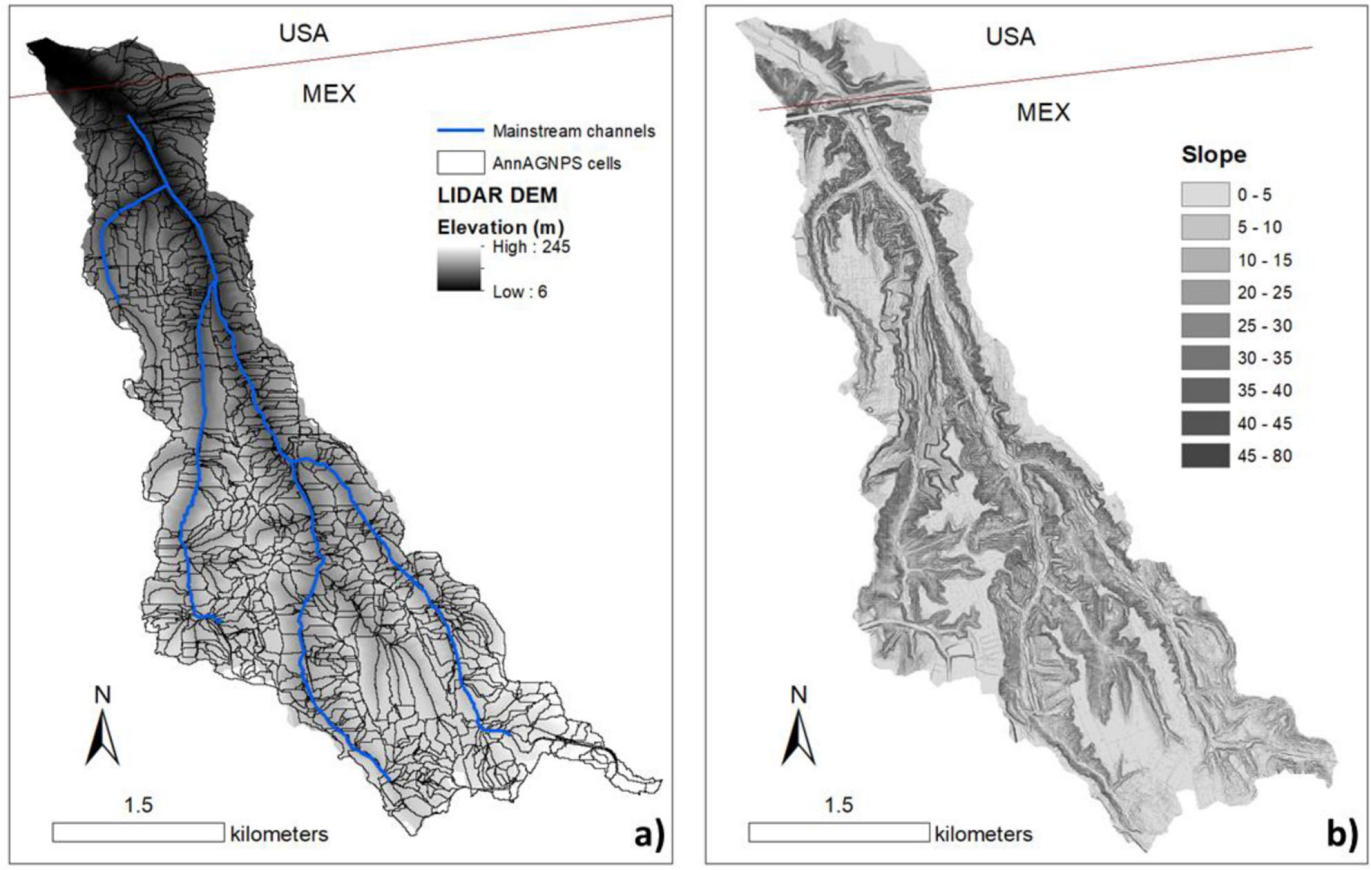
**(a)** AnnAGNPS cells, reaches, mainstream channels, and **(b)** slope gradient at the Los Laureles Canyon watershed (LLCW).

Application of AnnAGNPS can be challenging in a watershed with steep topography and sediment coarser than sand (Figure 4) because the model does not simulate mass wasting processes, only transports sediment up to coarse sand (2 mm), and is designed for mixed-use watersheds in agricultural areas. Mass wasting, including shallow landslides, was observed in the study area, and coarse sediment accounts for ~10–15% of the sediment in the traps at the watershed outlet (unpublished data). However, the valley floor at the base of the steep slopes that are most likely to experience landslides, has been graded and paved for roads on either side of the channel, which limit the transport of coarse material to the channel from landslides or other hillslope processes. Field observations suggest that landslides typically terminate on these flat road segments or other graded areas, and the coarse material that accumulates at the toe of a landslide is periodically cleared mechanically. In this scenario, we assumed that all coarse material is from the channel. The sediment load from channel erosion was taken from Reference [38] and added to the total modelled hillslope erosion. Lastly, the RUSLE equation to estimate sheet and rill erosion was designed for relatively flat agricultural hillslopes, and may not be valid for steep hillslopes (>30%) [39]. In this case, we assumed that the model application was valid in most of the study watershed because 87% of the total watershed area has a slope gradient of less than 30% (mean slope = 15%).

The spatial variability of topography, land cover, soils, and management properties within the catchment area was represented in the model by discretizing the watershed into cells that are relatively similar in slope, soils, and land use. A LIDAR-derived Digital Elevation Model (DEM) (3 m, horizontal resolution) of the LLCW (sponsored by the County of San Diego California, USA) was used as input for a topography-based method (TopAGNPS) [26]. The TopAGNPS method was used to (i) delineate surface flow paths, (ii) subdivide the total catchment area into sub-catchments (cells) along drainage segmentations, and (iii) estimate representative cell parameters, such as slope, area, and soil and management attributes. Cells sizes were based on user-defined values of the Critical Source Area (CSA), which is the minimum drainage area required to form a channel, and the Minimum Source Channel Length (MSCL), which prunes the channel network of channels shorter than the specified MSCL value.

To characterize the hillslope and reach units within the AnnAGNPS model, a CSA of 1 ha and a MSCL of 50 m were assigned, based on field observations, to characterize the hill-slope and reach units within the AnnAGNPS model. LLCW was discretized into 1147 sub-catchments (AnnAGNPS cells) and 462 channels (AnnAGNPS reaches). The cell sizes ranged from 9×l0^−6^ to 0.1 km^2^ (Figure 4).

TopAGNPS was also used to map Potential Ephemeral Gullies (PEGs) throughout the LLCW following the methodology described by Momm et al. [26]. This method provides an automated estimate of the downstream-most locations of knickpoints (i.e., PEGs), which are used within AnnAGNPS to calculate the length of ephemeral gullies in the landscape. The approach uses improvements on the EGEM described in Gordon et al. [40] and, more recently, revised by Bingner et al. [12]. In the model, the gullies are filled in once per year at the end of each wet season, which corresponds to observed management practices, but may under-represent the filling frequency on larger main roads that are typically filled between each storm event that generates gullies.

The watershed hydrology module of AnnAGNPS uses the SCS Curve Number method [27] to estimate storm event runoff from precipitation in each cell. The storm event water peak discharge and the time-to-peak are determined for the hydrograph at each reach section and at the watershed outlet, following the TR-55 [27] approach that utilizes the time of concentration for each cell and reach, determined with TOPAGNPS, total daily runoff determined from AnnAGNPS, and the storm type entered as an input parameter [12].

The spatial resolution of the DEM has little impact on AnnAGNPS runoff volume estimates [11,41], but soil erosion and sediment loads can change with DEM resolution, since resolution impacts slope [42]. LIDAR-derived 3-m DEMs should improve the model performance in the study watershed compared with applications that use more-commonly available 10 m or 30 m resolution DEMs. Initial AnnAGNPS reaches did not follow the road network, so the road segments were “burned” into the DEM by lowering the elevation on the DEM cells falling on roads by 1 m.

A land use map was created using Google Earth (11 November 2012, 2017 Digital Globe) imagery based on visual classification of seven categories (rangeland, highway road, paved residential roads, dispersed urban unpaved, unpaved rural roads, unpaved residential roads, and sediment basin). The accuracy of the land use data was validated by comparing land use categories with ground-based photography and field data collection. The land use map was overlain on the AnnAGNPS cells to populate the required hydrologic and management parameters needed for AnnAGNPS. The soils map was used to link the required physical variables from the SSURGO database to the model such as soil texture and erodibility, bulk density, and saturated conductivity. Tillage depth is the depth to an impervious soil layer, which limits the potential depth of gullies, and was determined as the depth of the gullies observed in the field [14]. The main equations solved within AnnAGNPS to estimate soil erosion are listed in Table 1. A detailed description of these equations are given by Bingner et al. [12].

See Gudino-Elizondo et al. [14] for a detailed description of the usage of these equations in the study watershed.

### 2.3. Model Calibration and Evaluation

The model performance metrics considered both graphical and statistical analyses to assess the best parameterization based on the coefficient of determination (R^2^), Root Mean Square Error (RMSE, Equation (1)), and the percent bias (PBIAS, Equation (2)), which are widely applied in hydrologic and erosion modeling [43].

#### 2.3.1. Runoff

Total and peak runoff measured at PT (outlet) for 14 storm events were used for model calibration. Manning’s *n* back-calculated from discharge measurements was consistent with literature values for “ordinary concrete lining” (0.013) [44] and with the channel condition at PT. Simulated total and peak discharge were then compared and evaluated using the RMSE, which is calculated by the equation below.

(1) $$RMSE=\sqrt{\frac{1}{N}\overset{N}{\underset{i=1}{\sum }}{\left(Observed\_i-Simulated\_i\right)}^{2}}$$

where *i* is the index of the storm events and *‘N’* is the number of events (14).

The selection of the AnnAGNPS model parameters to calibrate the runoff was based on the watershed characteristics, preliminary model runs, and literature values identified for each cell in the watershed [44,45] (Table 2).

#### 2.3.2. Sediment

Data on sediment removed from the sediment traps at the LLCW outlet (Figure 1) were used for calibration. Both upper and lower traps were excavated in the Spring and Fall of 2005, Winter 2006, and each Fall from 2007–2012 *(N* = 7). The sediment trap efficiency, or the proportion of the total sediment trapped in the sediment basin, was calculated based on Morris and Fan [46] and Urbonas and Stahre [47]. See Biggs et al. [33] for a detailed description.

Preliminary simulations suggested that the channel erosion module of AnnAGNPS resulted in excessive sedimentation in the channels, which was not observed in the field. We, therefore, calculated the simulated sediment load as the total amount of sediment by source (sheet and rill, and gully erosion) that makes it to the stream channel network (Figures 1 and 4), plus channel erosion estimated in previous work [38]. The load was compared to the total sediment load with the total amount of sediment being excavated from the sediment traps for specific dates. The stream channel network was defined as permanent channels within the watershed based on field observations and visual examination of these channels using high resolution imagery. Channel erosion estimates (t/yr) were taken from Taniguchi et al. [38], who calculated channel erosion from the difference between the cross sections observed in 2014 with the cross section under reference (pre-urban) conditions, which was divided by the time since urbanization. Taniguchi et al. [38] estimated that channel erosion accounted for 25% to 40% of total sediment yield to the estuary over 2002–2017. In this scenario, we estimate channel erosion by multiplying the hill-slope erosion estimated by AnnAGNPS by 0.33 and 0.67 to get channel contributions of 25% and 40% of total load, and adding that load from channel erosion to the hill-slope load to get the total load.

The data from the sediment trap, corrected for trap efficiency, were compared with AnnAGNPS simulation results of total load, including both hill-slope and channel erosion. Critical shear stress *τc* and sediment delivery ratio (SDR) were then calibrated to match the observed sediment yield at the LLCW outlet. An initial value of *τ*_*c*_ was set to 1.6 N-m^−2^ for sandy soils based on the average value from nine samples collected on the Lf soil type [14]. Initial values of *τc* for conglomerate soils were taken from USGS [37] dataset for fine cobbles (64 N·m^−2^) and were modified during calibration to τ^*c*^ = 32 N·m^−2^, which corresponds to very coarse gravel. The parameters used to calibrate sediment yield are presented in Table 3. The SDR for coarse soil formations (CfB and CbB) were calculated internally by the model. The SDR for the Lf type was set to 1 and based on field observations of extensive rill and gully formation, which results in the delivery of most sediment from sheet and rill erosion to the channel network.

The percent bias (PBIAS) was used as a measure of the average tendency of the simulated results relative to the observed data, which indicates over (positive PBIAS) or underestimation (negative PBIAS), respectively [48,49]. The PBIAS was calculated using the equation below.

(2) $$\text{PBIAS}=\frac{\overset{N}{\underset{i=1}{\sum }}\left(observed-simulated\right)\times 100}{\overset{N}{\underset{i=1}{\sum }}observed}$$

where *i* is the index of the storm events and *‘N’* is the number of events (14).

The measurements of sediment accumulation at the outlet provides an aggregate measure of sediment load for the watershed, but does not validate the spatial pattern of sediment load from different soil and land use units in the watershed. Grab samples of water were collected for suspended sediment analysis at 10 sites in the watershed during a large storm (81 mm, total depth) on 27 February 2017. All samples were collected over a 0.5 hour period, which corresponded to a period of maximum runoff. The observed SSC of the storm-water samples were then compared with the simulated AnnAGNPS SSC (SSC = storm event sediment mass/storm event runoff volume) to explore the influence of soil properties and land use on sediment production in the watershed. While SSC at a given location changes during an event, the samples were collected during similar hydrological conditions, and provide a snapshot of the spatial variability of SSC during an event. Table 4 summarizes the data type and parameters set for model calibration and evaluation.

We used the entire dataset of observations at the outlet, including annual sediment accumulation in the traps (N = 6) and event runoff (N = 14), for model calibration due to the small number of observations. Use of an entire dataset for calibration is consistent with other AnnAGNPS applications in Mediterranean environments such as References [50,51].

### 2.4. Scenario Analysis

We evaluated the impact of paving roads on runoff and sediment yield using the calibrated model. The simulation paved only those roads in the AnnAGNPS cells that generated 50% of the total sediment yield at the LLCW scale (hotspots) under current conditions. For the scenario analysis, we assumed that the *CN* is the same for all paved roads *(CN* = 98), and that gully sediment yield is zero since gully erosion occurred solely on the dirt road network within the LLCW [9,14]. Composite curve numbers were calculated for unpaved and paved conditions following Gudino-Elizondo et al. [14]. The scenarios were run for 2004–2017 and the impact on sediment and runoff were determined by the change in simulated sediment load, and total and peak runoff between the current conditions and the paving scenario. The change in total and peak discharge were calculated for the largest 14 storm events (event-total precipitation ranging from 28 to 81 mm).

## 3. Results

### 3.1. Rainfall Data

**Figure 5.**
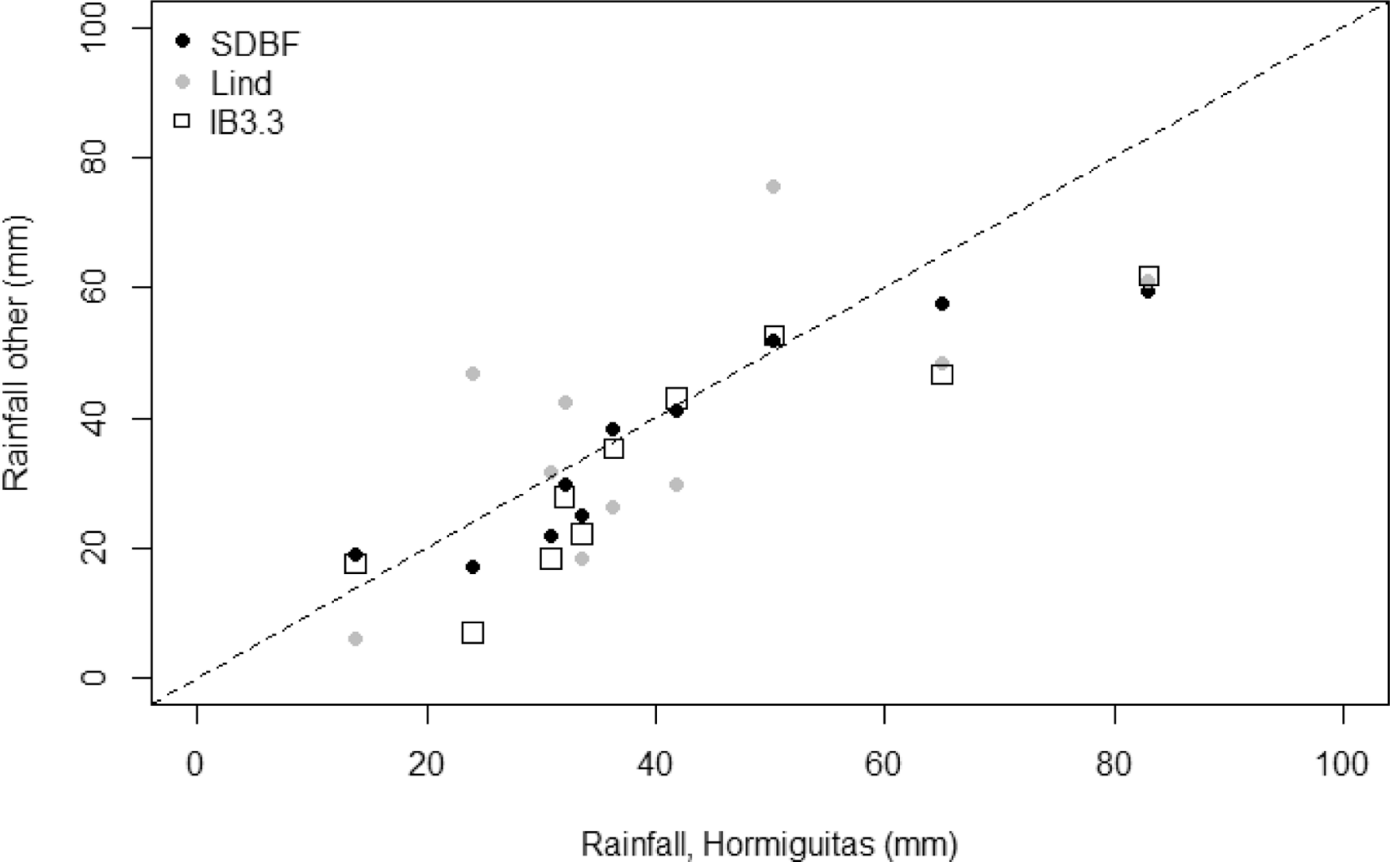
Event-total precipitation at the Hormiguitas rain gauge (RG.HM) versus three other nearby stations (see Figure 1). The dashed line is the 1:1 line. Taken from Biggs et al. [33].

Total event rainfall at the rain gauge in LLCW (RG.HM, Figure 1) correlated closely with daily rainfall at nearby stations in the United States (Figure 5). For the events when rainfall data were recorded for the LLCW watershed at RG.HM (2013–2017), the gauge at San Diego Brownfields (SDBF) has the highest correlation coefficient and smallest RMSE out of the stations with good data availability. Rainfall at RG.HM was higher than that at all other stations for larger events (> 60 mm), but matched the SDBF data well for rainfall between 10 and 50 mm (Figure 4). The SDBF gauge had a higher correlation coefficient and lower error compared to stations closer to LLCW in the Tijuana Estuary (IB3.3). Therefore, SDBF was used to estimate rainfall in LLCW for years when no data was available at RG.HM.

### 3.2. Rainfall-Runoff Relationships

**Figure 6.**
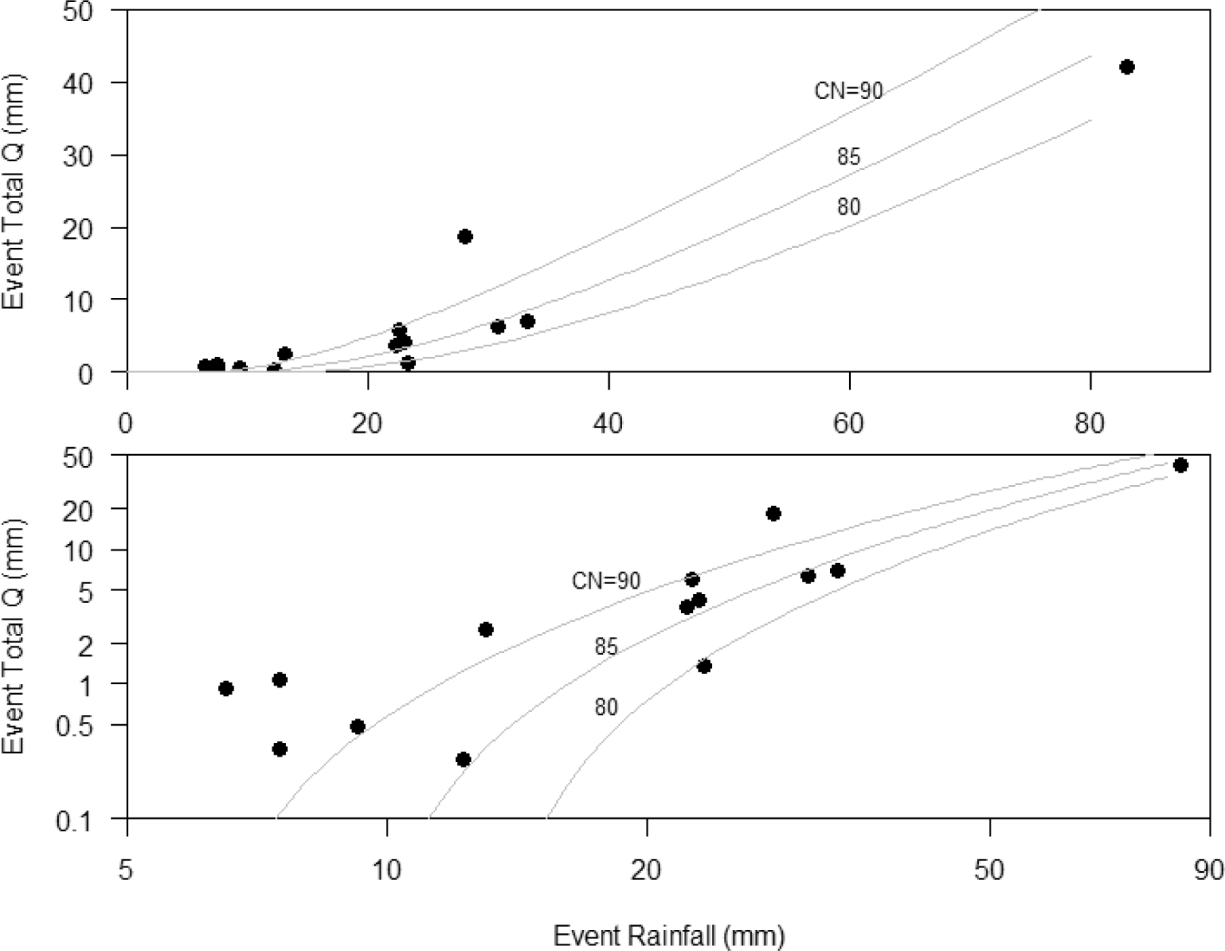
Rainfall-runoff relationship for all observed storm events, with several SCS CN rainfall-runoff relationships, in non-log (top) and log-log (bottom). Taken from Biggs et al. [33].

Event-total rainfall for the 14 events with rainfall (P) and runoff (Q) data ranged from 7 to 83 mm (Table 5). The event-wise runoff coefficients (Q:P) ranged from 0.02 to 0.67. Event-total runoff increased with event-total rainfall and fits a watershed-mean SCS CN of 80–90 (Table 5 and Figure 6). The highest SCS CN occurred for the smallest events and CN generally decreased with the event size (Figure 6). This was consistent with runoff production from surfaces with low infiltration capacity during small events, and from all surfaces, including those with high infiltration capacities, during large events. The largest event (rainfall 81 mm) has a runoff coefficient of 0.51, where most points fell between SCS CN 80 and 90 (Figure 6), which is consistent with literature values for partially urbanized land cover [45]. Thus, no adjustments were needed for the CN as the fit was adequate with the observed storm-wise rainfall-runoff relationships (Figure 6). The 24-hour rainfall distribution used for most of the simulated storms was type II [27] because it is representative of semi-arid regions of South-western USA, and matches the most frequent storm type calculated using rain gauge measurements in the LLCW [33]. Some storms were assigned different storm types based on their rainfall distribution compared to SCS storm types.

**Figure 7.**
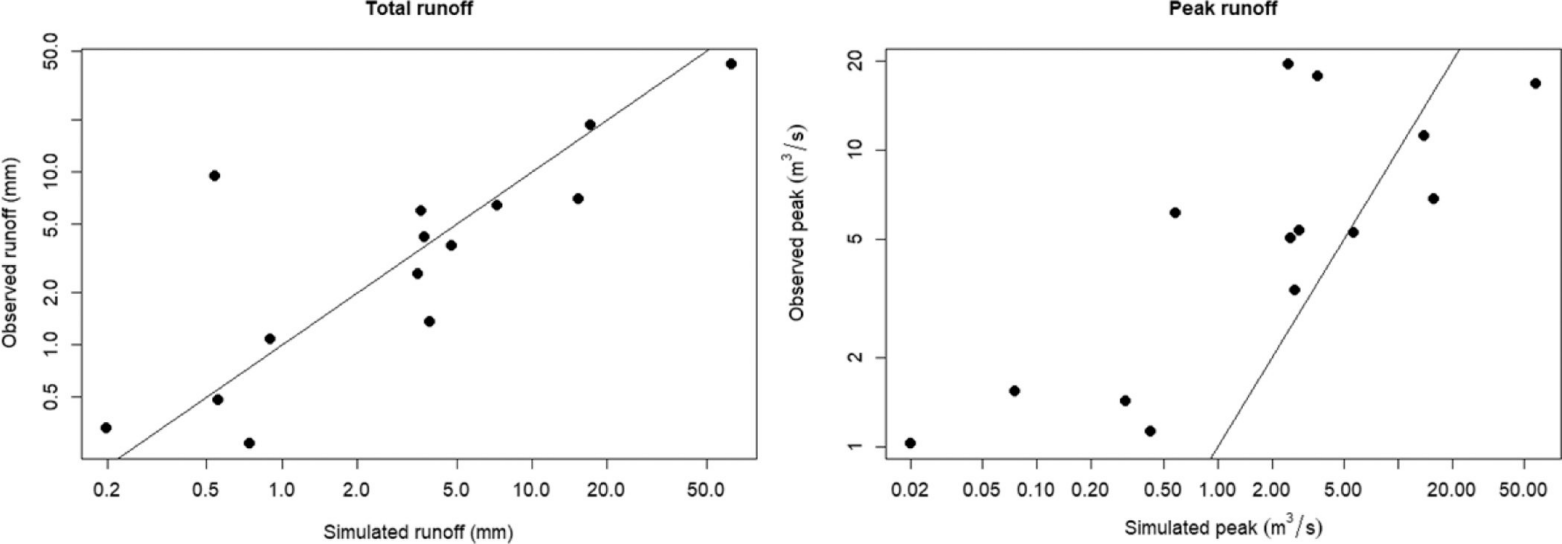
Relationship between observed and simulated total (a, mm per storm) and peak (b, m^3^/s) runoff. The solid lines are the 1:1 lines.

The RMSE of the simulated storm-wise runoff was 6.6 mm (89% of mean), and 13 m^3^·s^−1^ (177% of the mean) for peak runoff. The RMSEs were notably influenced by a single large storm of 81 mm total-event precipitation (27 February 2017, Table 5). RMSE without that large storm was 3.6 mm (75% of the mean) for total runoff, and 6.9 m^3^·s^−1^ (105% of the mean) for peak runoff. The AnnAGNPS model was most accurate for medium-sized events (event precipitation between 2 and 20 mm, Figure 7), which are the most frequent events. Therefore, we did not calibrate the model to minimize the error. Peak discharge was generally underestimated for small storms and overestimated for large storms [33].

### 3.3. Simulated Sediment Production

**Figure 8.**
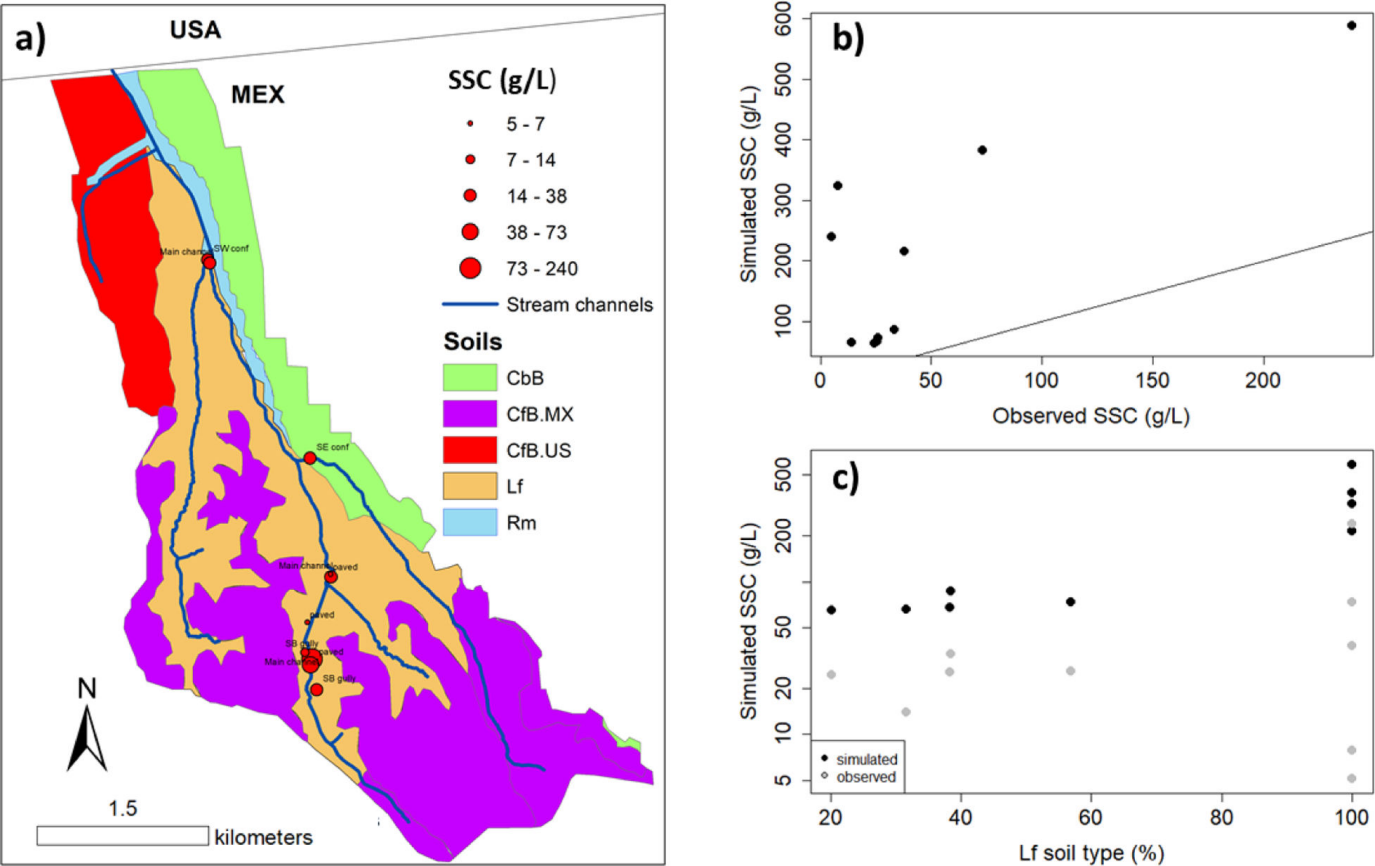
**(a)** Geographic location of storm-water samples, suspended sediment concentration (SSC), and soil types along the LLCW. **(b)** Relationship between observed and simulated SSC (the black line is the 1:1 line) and **(c)** relationship between Las flores (Lf) soil fraction and simulated and observed SSC.

The SSC samples were collected from sub-water sheds with different soil characteristics. Fractional covers of soil types in the sub-watersheds draining to each SSC sample location vary from 20% to 100% erodible, non-cobbly soils (Lf soil type) (Figure 8). The observed SSC of the storm-water samples correlated with the AnnAGNPS-simulated SSC (Figure 8b), and modelled SSC correlated with the fraction of the sub-watersheds covered by Lf (Figure 8a,c), which highlight the influence of soil properties on the modelled sediment production. The modelled SSC values were higher than the observed SSC values (mean model = 210.7 g/L, mean observed = 48.7 g/L, RMSE = 203.5 g/L) because the grab samples were not all taken at the time of the peak discharge. No samples were available for areas drained by cobble and gravel soils in the southern and northern parts of the watershed.

### 3.4. Sediment Budget

**Figure 9.**
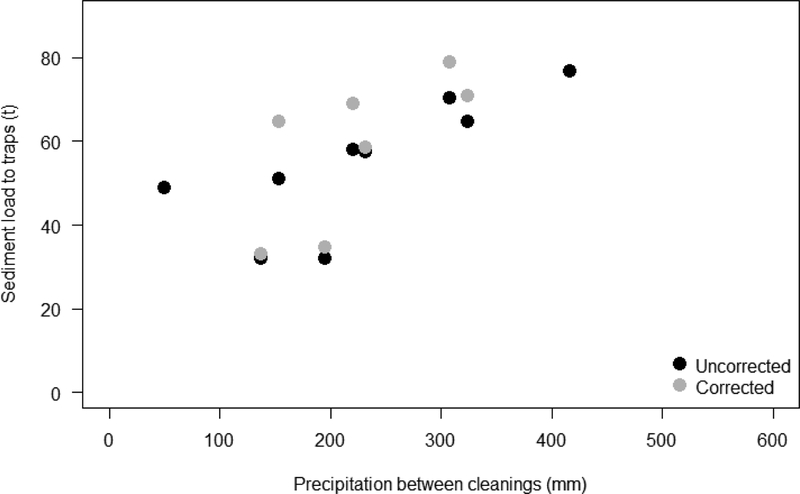
Total sediment removed from the Los Laureles Canyon traps in the United States versus total annual precipitation between removal events from 2005 to 2012. The uncorrected amount of sediment removed is in black and the load corrected for trap efficiency is in grey. Annual precipitation is from the San Diego Brownfield station. Modified from Biggs et al. [33].

The annual trap efficiency varied from 0.79 to 0.98, and was 0.89 for the cumulative mass removed over 2006–2012 [33]. Uncertainty in sediment trap efficiency calculation, in particular the gradual filling of the traps during the year and subsequent decrease in trap efficiency, may have caused underestimation of sediment load at the traps (Table 6). Total annual sediment accumulation in the traps correlates with annual precipitation at the SDBF station (Figure 9).

**Figure 10.**
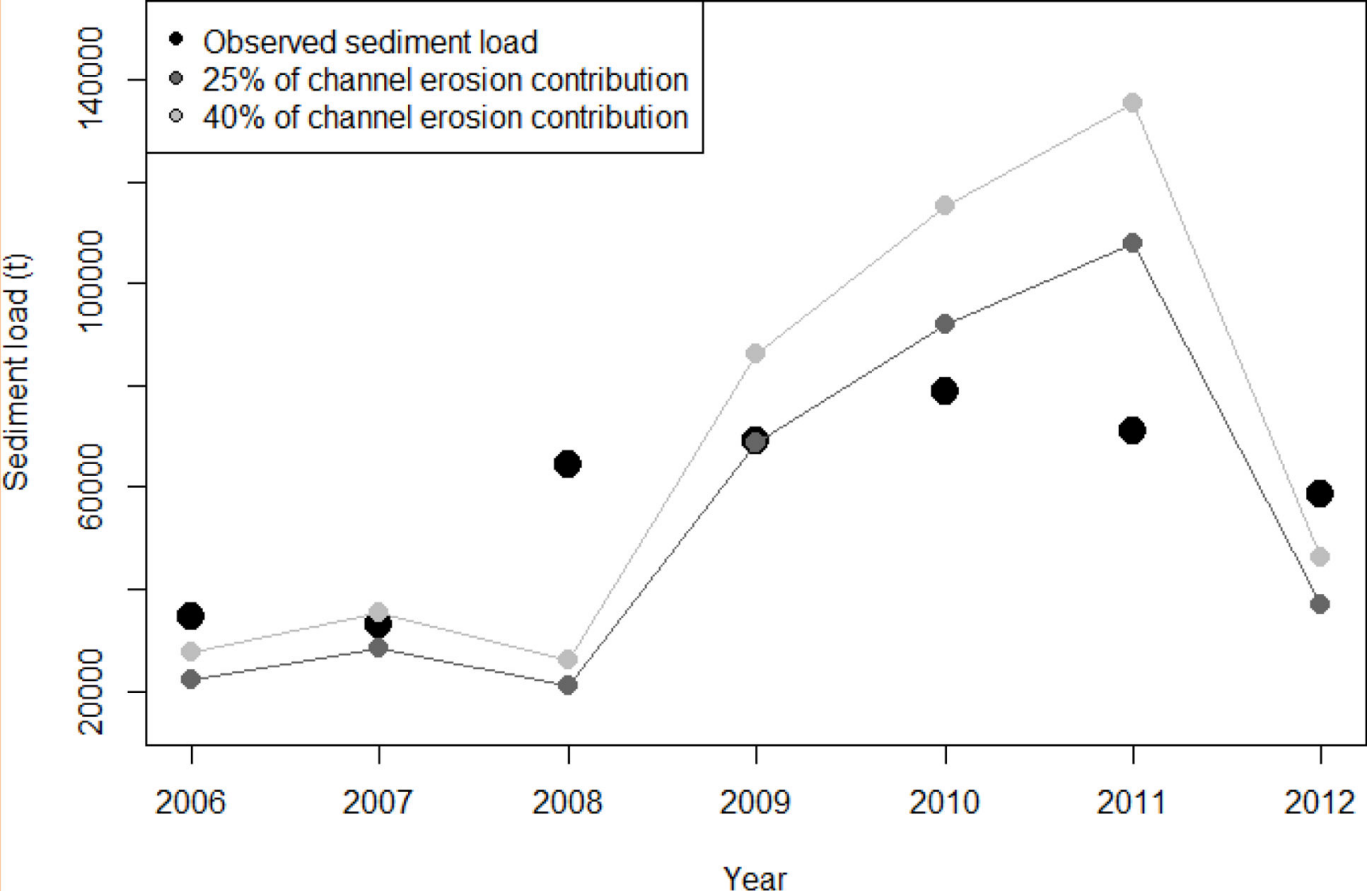
Time series of the relationship between observed and simulated annual sediment load at the LLCW outlet, assuming a channel erosion contribution of 25% and 40% of the total hill-slope sediment production.

Total modelled sediment load correlates with the sediment load observed at the sediment trap (Figure 10), with the following errors: (i) pBIAS25% = 8.1, RMSE25% = 24,115 t (41% of the mean) considering a channel erosion contribution of 25% of the total hill-slope sediment production, and (ii) pBIAS4o% = 1^.4, RMSE4o% = 32,570 (55% of the mean) considering a channel erosion contribution of 40%. These model efficiencies were moderate-to-good according to other studies [44,49,52] reporting relatively similar values.

The default values of τ_*c*_ for conglomerate soil types (CfB and CbB) (τ_*c*_ = 64 N-m^−2^) resulted, on average, in underestimation of the total sediment load at the outlet, so it was changed during calibration to τ_*c*_ = 32 N-m^−2^ to fit better with the observed sediment yield in the sediment traps. The τ_*c*_ value set in the calibrated model corresponds to very coarse gravel [37], which was consistent with the observed particle sizes of gravelly and cobbled soils in the LLCW [33].

**Figure 11.**
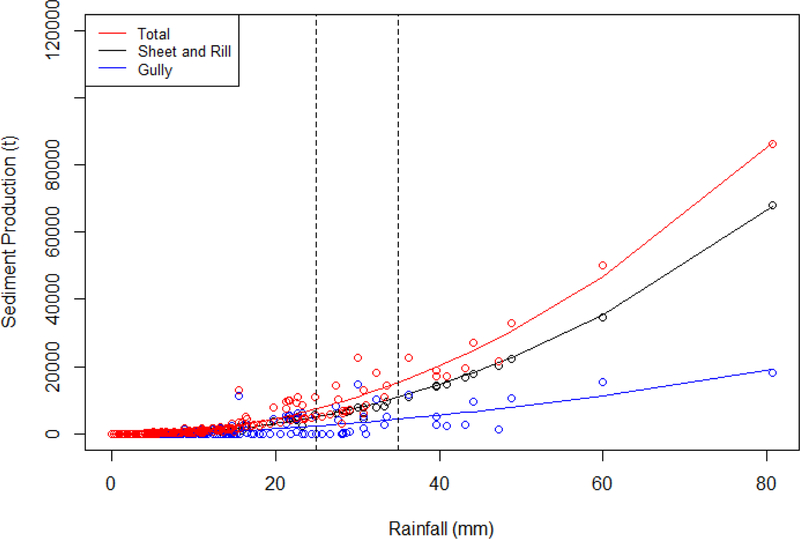
Simulated sediment load by erosion processes in LLCW. The vertical dashed lines indicate the range of the rainfall threshold for gully erosion observed in the field during 2013 to 2018.

Precipitation correlates with simulated sediment production from sheet and rill erosion, gully erosion, and total sediment yield, while sediment production by sheet and rill erosion correlates more closely with rainfall than gully sediment production does (Figure 11). A minimum precipitation threshold (−25–35 mm) for gully initiation was reported by Gudino-Elizondo et al. [9], which is consistent with the significant contribution by gullies to the total sediment production for those storm events with precipitation greater than 25 mm (Figure 11).

**Figure 12.**
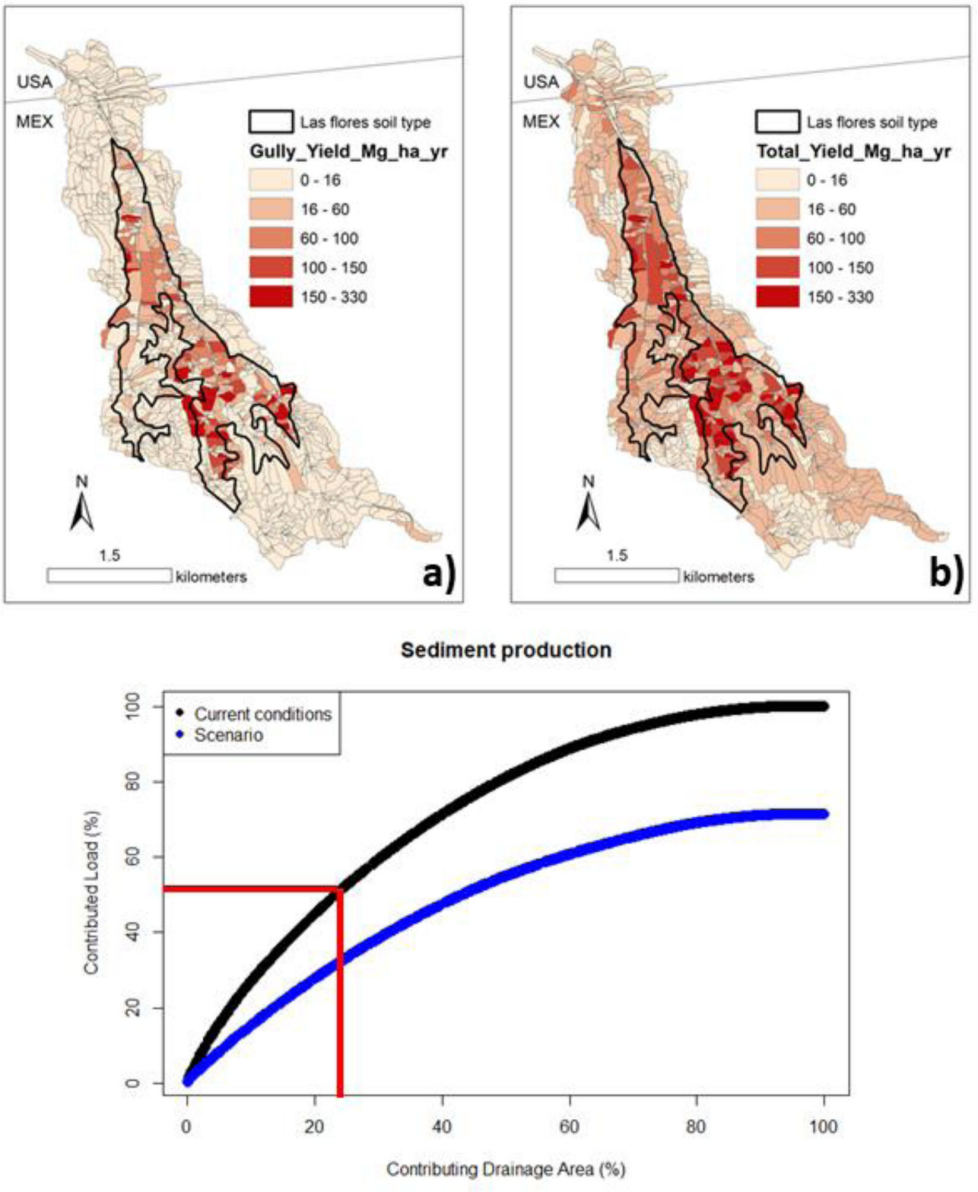
**(a)** Sub-watershed sediment yield by gully erosion, **(b)** Total sediment yield by sub-watershed within the Los Laureles Canyon watershed and **(c)** total sediment production by contributing the drainage area under the current conditions and road-paved scenario.

Simulated sheet and rill erosion was the dominant erosional processes within the LLCW (Figure 12), which was also reflected in the event-wise rainfall-sediment relationships (Figure 11), especially for larger events. Total sediment load at the sub-watershed scale (AnnAGNPS cells) was dominated by cells characterized by sandy soil types (Lf) on steep slopes, which show evidence of frequent rill and gully formation (Figure 12).

The observed sediment in the trap is finer (higher silt fraction) than both the hill-slope sediment and the AnnAGNPS-simulated sediment load (Figure 2). This suggests that either more sand is being retained in storage on the hill-slopes and in the channel than is simulated by the model, or that silt is preferentially eroded from soils that have a mixture of silt and sand, or that soils with high silt fraction contribute more to the load than is being modelled. The particle size in the Mexico sediment trap is coarser than the US sediment trap, which suggests either retention of sand in the channel downstream of the Mexico sediment trap, or high loads of silt from the sub-watershed outside of the Mexico sediment trap sub-watershed.

### 3.5. Scenario Analysis

Half of the simulated sediment load at the watershed scale is generated by only 23% of the total watershed area under current conditions (red lines in Figure 12). These cells are hotspots of sediment production and, pending validation of erosional severity with additional field observations, could be prioritized for management activities to reduce sediment production at the watershed scale.

The model scenario suggests that, on annual average, paving all the roads in the hotspots would reduce sediment production by 30% (Figure 12). However, storm-wise total runoff increases by an average of 10%, and peak runoff increases from 1.6% to 21% (Table 7). The projected peak discharge increased the most for the medium-sized events (40–49 mm, two-year recurrence interval), and not for the largest event (81 mm, 25-year recurrence interval), which suggests that paving roads in hotspots could be suitable for the study watershed without increasing peak discharge for the largest events. This may be the most responsible factor for flood damage.

## 4. Discussion

AnnAGNPS simulated total water and sediment load with satisfactory agreement with observed total event runoff and sediment yield in the LLCW. The simulated total runoff and peak discharge were more accurate for medium-sized events (event precipitation between 2 and 20 mm, Figure 7), which are the most frequent events in the region. The model generally underestimated peak discharge for small storms and overestimated peak discharge for large storms, which is partly due to underestimation of the peak rainfall rates of small storms and overestimation of the peak rainfall rates of large storms by the SCS Storm type model [33]. Further research on the impact of paving on peak discharge for a range of storm sizes and sequences is needed.

The AnnAGNPS model satisfactorily simulated ephemeral gully erosion rates in the LLCW at the neighborhood scale [14], which helped identify parameter values for use at the LLCW scale. The model performed well compared with other models applied in semi-arid environments [23,30,49,51,53] which supported its use for runoff and sediment budgets in this watershed. However, uncertainties in soil-resistance-to-erosion parameters, especially critical shear stress for cobbled soils, may affect sediment production by gully erosion. This suggests that more field and laboratory data are necessary to have more accurate sediment yield estimates at the watershed scale.

The SSC from 10 grab samples (Figure 8c) collected during the largest storm event correlated with modelled SSC, which suggests the model represented spatial variations in sediment production within the watershed. Modelled erosion was sensitive to the fraction of highly-erodible Lf soil type, which generated 61% of the total sediment load. Most of the AnnAGNPS cells that contribute significantly to the total sediment load (hotspots) had both highly erodible soils (Lf) and steep slopes (>30%) that encourage gully sediment production (Figure 12). No SSC samples were available for areas drained by cobbled and gravel soils in the northern part of the watershed, so future work should include more grab samples from sub-watersheds draining cobbled soils.

The RMSE of the AnnAGNPS model for sediment load was 41% and 55% of the mean value considering 25% and 40% of channel erosion contribution, respectively. Our observed values of sediment load at the outlet likely underestimate the total load because our method for calculating trap efficiency does not account for a reduction in trap efficiency as the trap fills during the wet season. This underestimate is likely largest during wet years when the trap is full at the end of the season, so future research will explore the impact of reduction in available trap capacity on trap efficiency and estimated sediment load. Channel evolution is also not well characterized by the AnnAGNPS model, which reduces the performance of the model to simulate the observed behavior of the system. Taniguchi et al. [38] noted that urbanization caused extreme channel enlargement in the LLCW, which suggests the necessity to implement and couple a more sophisticated channel evolution model to AnnAGNPS such as the channel evolution computer model (CONCEPTS) [54,55] to better simulate the sediment production at the LLCW scale.

Simulated gully erosion represented approximately 16% and 37% of total sediment production considering 25% and 40% of channel erosion contribution, respectively. This was relatively close to other estimates for human-disturbed watersheds. Bingner et al. [56] reported that ephemeral gullies were the primary source of sediment (73% of the total) in agricultural settings within the Maumee River basin, USA. De Santiesteban et al. [57] found that ephemeral gullies contributed 66% to total soil loss in a small agricultural watershed. Taguas et al. [23] found that contribution of gully erosion to the total soil loss varies substantially depending on the management, on average, from 19% to 46% under spontaneous grass cover and under conventional tillage management. Previous studies reported gully erosion in agricultural and partially urbanized watersheds even though gully erosion can be a significant, and often neglected, portion of the sediment budget. We likely underestimate gully contribution since the model assumes gullies on roads are filled only once per year, while field observations suggest that main roads are repaired several times per year, after every storm that generates gullies. Our estimates of gully contribution are also sensitive to the mapped distribution of fine-textured soils that generate most of the gullies. Future research could refine the soils map and test for the sensitivity of gully filling frequency on the gully contribution.

Our estimate of the contribution of channel erosion to the sediment load (25%–40%) is smaller than other studies, which report channel contributions ranging from 67% [58] to 85% [59] of the total sediment yield in urban areas. The relatively large contribution from hill-slope sources in our study area is likely due to persistent soil exposure and erosion, including vacant lots and unpaved roads, that characterizes urbanization in Tijuana [8] and possibly other cities in developing countries.

In our watershed study, 50% of the sediment production under current conditions is generated only from 23% of the watershed area. Paving all the current unpaved roads in these “hot spots” would reduce sediment production by 30% compared to the current conditions, but it would also increase total discharge by 2%–17% and peak discharge by 2%–21%. The smallest increase in peak was for the largest event, which suggests that the impacts of paving may be small for the events that cause the most flood damage. This is consistent with other studies that document proportionately large impacts of urbanization on the smallest events, and declining impact for larger events [60], even though more complete documentation of the impact of paving for a range of storm sizes under different antecedent moisture conditions is necessary.

This investigation highlights the necessity to implement management activities to mitigate soil erosion such as stabilization of unpaved roads and other management activities (i.e., revegetation, sediment basins, channel stabilization, etc). Future studies should evaluate the uncertainty of the model-estimated parameters as well as implications in scenario analysis [14], which are critical for proper sediment management in the LLCW and potentially in other rural urbanizing watersheds, particularly those in developing countries. Our study highlights the relative importance of various erosion processes, and also key uncertainties for future investigation.

## 5. Conclusions

Urban development has significant impacts on watershed sediment production in a developing country context. Management activities, especially the practice of filling gullies with poorly-consolidated materials, represent a persistent source of sediment in the watershed. Simulated total runoff correlated well with the observed data whereas simulated peak discharge was best predicted for medium-sized events. Simulated gully erosion contributed significantly to the total sediment load at the watershed scale (20% to 26%, annual average), even though most (40%–50%) of the total load was from sheet and rill erosion. Hotspots of erosion cover 23% of the total catchment area but generate 50% of the total sediment yield, and occur on steep slopes on highly erodible soils. This investigation highlights the necessity to implement management activities to mitigate soil erosion such as asphalt or other stabilization measures on unpaved roads, as well as other management activities (i.e., revegetation, sediment basins, channelization, etc). The scenario analysis showed that paving roads in the hotspots reduced total sediment production by 30%, but may increase peak discharge moderately (2%–21%) at the outlet. Any mitigation activity in the watershed that includes road paving needs to consider the potential impacts on downstream communities and channel erosion. Future studies for improving model calibration, and evaluating more mitigation scenarios are critical for proper sediment management in the LLCW and potentially in other urbanizing watersheds, particularly those in developing countries. Our maps of the spatial distribution of sediment yield are uncertain due to the coarse resolution of land use and soil properties for small sub-watersheds (AnnAGNPS cells), and possible overestimation of sheet and rill erosion on steep slopes. Future research should include more detailed spatial information on soil properties to improve parameters’ estimates, which would improve the accuracy of model simulations under current conditions and in various management scenarios.

## Figures and Tables

**Table 1.** Equations and parameters used to simulate soil erosion in the AnnAGNPS model.

Module	Equation
Sediment yield by sheet and rill erosion	${\text{S}}_{\text{y}}=0.22\times {\text{Q}}^{0.68}\times {\text{q}}_{\text{p}}{}^{0.95}\times \text{KLSCP}$
Gully width	W = 9.0057 × (Q_p_ × S)^0.2963^
Head-cut migration erodibility coefficient	$\text{Kh}=0.0000002/\sqrt{\tau c}$

Annotation: S_y_ = sediment yield by sheet and rill erosion (Mg/ha): Q = surface runoff volume (mm). qp = peak rate of surface runoff (mm/s) and K, L, S, C, P are RUSLE factors. W = gully width (m): Q_p_ = peak discharge at the gully head (m^3^/s). S = the average bed slope above the gully head (m/m). K_h_ = head-cut migration erodibility coefficient (m^3^/s/N): *τ*_*c*_ = the critical shear stress (N/m^2^).

**Table 2.** Final AnnAGNPS runoff parameters values used to calibrate runoff.

	Range Land		Disperse Urban Unpaved		Non-Urban Unpaved	Paved Residential Roads		Unpaved Rural Roads	Unpaved Residential Roads		MX Sediment Basin
Hydrologic soil group	B	D	B	D	D	B	D	D	B	D	D
Percent watershed area	2	30	1	11	5	3	10	1.3	4	32	0.7
Curve number	77	88	88	94	98	98		89	82	89	98
Manning’s *n* of overland flow	0.13		0.07		0.01	0.01		0.07	0.03		0.01

**Table 3.** AnnAGNPS soil erosion parameters used to calibrate the watershed scale model for sediments.

	Lf	CbB	CfB.MX	CfB.US
Critical shear stress (N·m^−2^)	0.1	32	32	32
Tillage depth (cm)	60	60	60	60
Sediment delivery ratio	1	Internally calculated	Internally calculated	Internally calculated
USLE *K* (t ha hr)/(ha MJ mm)	0.036	0.006	0.048	0.048
Hydrologic soil group	D	B	D	D

**Table 4.** Field data collection and time periods for model calibration.

Type of Data	Dates	AnnAGNPS Parameters	Model
Water discharge	14 events (2013–2017)	Storm type, Manning’s *n*	Calibration
Sediment traps	7 excavation periods	SDR, τ_c_, tillage depth, USLE-K	Calibration
SSC (grab samples)	1 event (Feb 2017)	None	Evaluation

**Table 5.** Summary of storm events used for model calibration.

Event	Rainfall (mm)	Peak Discharge (m^3^/s)		Total Runoff (mm)	
		Observed	Simulated	Observed	Simulated
28 February 2014	12.25	1.13	0.43	0.27	0.74
1 March 2014	7.50	1.54	0.08	0.33	0.20
2 March 2014	7.50	6.14	0.58	1.08	0.90
1 March 2015	23.25	3.36	2.69	1.36	3.91
2 March 2015	9.25	1.43	0.31	0.48	0.56
15 May 2015	22.50	19.46	2.46	5.93	3.62
15 September 2015	30.75	5.27	5.69	6.40	7.27
5 January 2016	22.25	17.72	3.58	3.76	4.79
6 March 2016	6.50	1.03	0.00	0.93	0.01
7 March 2016	23.00	5.07	2.55	4.23	3.74
19 January 2017	13.00	5.37	2.85	2.57	3.51
20 January 2017	28.00	6.86	15.91	18.66	17.24
17 February 2017	33.25	11.16	13.88	7.03	15.31
27 February 2017	81.00	16.69	58.23	42.07	63.12
TOTAL	320	102	109	95	125
RMSE		13		6.6	

**Table 6.** Simulated annual sediment yield and total observed (in tons) at the watershed outlet by the erosion process. The range values for channel contribution and total yield assumes channel erosion is 25% (minimum value) and 40% (maximum) of the total simulated results from AnnAGNPS.

Year	Rainfall (mm)	Sheet and Rill (t)	Gully (t)	Channel (t)	Total (t)	Observed (t)	Ratio of Simulated to Observed
2006	193	8483	8143	5487–11140	22113–27766	34642	0.64–0.80
2007	136	15257	6022	7022–14257	28301–35536	33079	0.85–1.07
2008	154	10204	5518	5189–10534	20911–26257	64580	0.32–0.40
2009	218	38058	13555	17032–34580	68645–86193	68949	1.00–1.25
2010	298	48347	20669	22775–46240	91791–115256	78935	1.16–1.46
2011	323	51752	29273	26738–54287	107763–135311	70965	1.51–1.90
2012	234	16992	10797	9170–18619	36960–46408	58513	0.63–0.80
Mean	222	27013	13425	13345–27094	53783–67532	58523	0.88–1.10

**Table 7.** Rainfall and simulated increases in peak and total discharge volume at the outlet under current and scenario (hotspot paving) conditions for the 14 largest storm events.

Date	Rainfall (mm)	Increase in Peak Runoff (%)	Increase in Total Runoff (%)	Decrease in Sediment Load (%)
27 February 2017	80.75	1.63	1.98	29.97
27 February 2004	59.90	10.19	9.82	16.59
7 December 2009	48.80	17.78	15.28	19.32
22 December 2016	47.25	10.34	9.64	37.73
13 December 2012	44.20	21.09	17.68	58.18
17 December 2008	43.20	8.92	8.71	23.84
22 December 2010	40.90	9.13	8.86	35.91
27 October 2004	39.60	7.82	7.40	24.61
19 February 2007	39.60	16.77	14.8	7.36
17 February 2017	31.00	6.07	5.12	16.01
26 February 2011	30.00	7.31	6.36	15.92
3 January 2005	26.70	5.27	5.23	15.86
4 January 2005	24.40	4.50	4.39	26.25
11 January 2005	23.60	3.78	3.58	36.24
Min	23.6	1.63	1.98	7.36
Max	80.75	21.09	17.68	58.18
